# A Supraventricular Tachycardia: What Is It? Where Should One Ablate?

**DOI:** 10.19102/icrm.2017.080402

**Published:** 2017-04-15

**Authors:** Amir Rahim Chaudhari, Brian Olshansky

**Affiliations:** ^1^Mercy Medical Center - North Iowa, Mason City, IA

**Keywords:** Ablation, pacing, supraventricular tachycardia, ventricular tachycardia

## Abstract

During electrophysiology testing, cessation of atrial pacing resulted in a tachycardia with 2:1, that changed to 1:1 atrioventricular conduction after a premature ventricular contraction. The mechanism and the location to ablate are discussed.

## Case presentation

**Figure 1:**
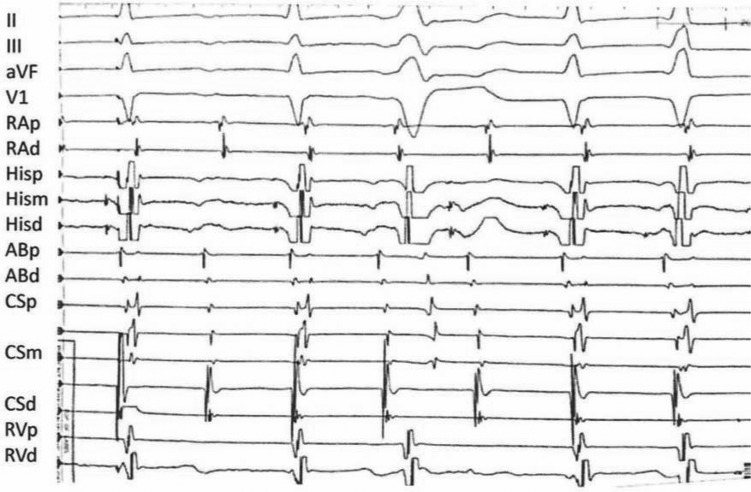
Distal coronary sinus pacing results in tachycardia with 2:1 atrioventricular conduction. 1:1 conduction occurs after a premature ventricular contraction. Shown here are electrocardiogram leads II, III, aVF and V1 and intracardiac electrograms. RA: right atrium; His: His-bundle; AB: ablation catheter; CS: coronary sinus; RV: right ventricle; p: proximal; m: middle; d: distal.

A patient with recurrent, symptomatic supraventricular tachycardia came to the electrophysiology laboratory for ablation. Tachycardia was initiated with atrial pacing from the distal coronary sinus **(Figure 1)**. 2:1, which became 1:1 atrioventricular (AV) conduction following a premature ventricular contraction (PVC), was induced reproducibly.

### The challenge

What is the tachycardia? Why did AV conduction change after a PVC? What explains coronary sinus activation? Where is the successful ablation site?

## Discussion

During electrophysiology testing, distal coronary sinus pacing resulted in tachycardia with two atrial activations before a ventricular activation. This tachycardia, however, was not atrial tachycardia or flutter; rather, it was typical AV nodal re-entry tachycardia. Ablation was performed successfully by a single radiofrequency lesion targeted at the slow pathway located at a typical posterior septal site, and not at an early atrial site.

During tachycardia, the earliest retrograde activation was recorded at the His electrogram, and then the proximal coronary sinus (CS). Earlier-than-expected atrial activation at a distal CS site is consistent with conduction up the fast pathway on the anterior septum transversing Bachmann’s bundle. This effectively (but not completely) rules out typical isthmus-dependant atrial flutter.

The 2:1 block is above, below or at the His-bundle in the AV node extensions as they enter into the His-bundle, but slow pathway antegrade and fast pathway retrograde activation persist. The tachycardia continues in spite of the PVCs, but the PVCs make the ventricles refractory so that His-bundle activation cannot proceed to the ventricles. This appears to reset the 2:1 timing, and allows for conduction to proceed through the extension, with a slightly slower tachycardia, but with 1:1 activation.

Concordant AV activation is not necessary for atrioventricular nodal re-entrant tachycardia (AVNRT). Block can occur either at or below the His-bundle. Sophisticated electroanatomical mapping techniques would not necessarily help to better understand the tachycardia, or lead to a more successful ablation.

As part of this initial installment of the new *Case Unicorns* section of *The Journal of Innovations in Cardiac Rhythm Management,* we welcome discussions from Drs. Katritsis, Verma, Knight, Jackman, Tchou and Efimov regarding this case.

